# Efficient photocatalytic degradation of bisphenol A on 2D-3D spherically hierarchical structure Zn_5_In_2_S_8_

**DOI:** 10.3389/fchem.2024.1519370

**Published:** 2025-01-16

**Authors:** Zongwen Zhang, Yi Zhang, Huili Han, Riyadh Ramadhan Ikreedeegh, Syed Shoaib Ahmad Shah, Muhammad Tayyab

**Affiliations:** ^1^ Analysis and Testing Center, Xinyang Normal University, Xinyang, Henan, China; ^2^ Key Laboratory of Green and Precise Synthetic Chemistry, Department of Chemistry, Ministry of Education, Huaibei Normal University, Huaibei, Anhui, China; ^3^ Collaborative Innovation Center of Henan Province for Energy-Saving Building Materials, Xinyang Normal University, Xinyang, Henan, China; ^4^ Department of Analysis and Quality Control, Sarir Oil Refinery, Arabian Gulf Oil Company, Benghazi, Libya; ^5^ Libyan Advanced Center for Chemical Analysis, Libyan Authority for Scientific Research, Tripoli, Libya; ^6^ Department of Chemistry, School of Natural Sciences, National University of Sciences and Technology, Islamabad, Pakistan; ^7^ Institute of Materials Research, Tsinghua Shenzhen International Graduate School, Tsinghua University, Shenzhen, Guangdong, China

**Keywords:** photocatalysis, Zn-in-S, photocatalytic degradation, bisphenol A, visible light

## Abstract

Bisphenol A (BPA) poses a significant environmental threat due to its widespread use as an industrial chemical and its classification as an environmental endocrine disruptor. The urgent need for effective BPA removal has driven research toward innovative solutions. In this study, we present the synthesis and application of a novel 2D-3D spherically hierarchical Zn_5_In_2_S_8_ (ZIS) photocatalyst for the photocatalytic degradation of BPA under visible light for the first time. Compared to the conventional g-C_3_N_4_ photocatalyst, ZIS exhibits enhanced optical and electrical properties, leading to remarkable photocatalytic performance, with an apparent reaction rate constant of 2.36 h⁻^1^, 6.56 times greater than that of g-C_3_N_4_. This efficacy allows for the degradation of 99.9% of BPA in just 2 h. The photocatalytic mechanism of ZIS was elucidated through various material characterizations and photoelectrochemical assessments, demonstrating improved light absorption and efficient charge separation as key factors facilitating BPA degradation. Notably, ZIS maintains high photocatalytic activity and stability over multiple cycles, indicating its potential as a sustainable photocatalyst. These findings not only contribute to the development of efficient photocatalysts for environmental remediation but also underscore the significant role of Zn_5_In_2_S_8_ in photocatalysis and solar energy conversion.

## 1 Introduction

The rapid advancements in science and technology have transformed society, bringing substantial material wealth but also posing significant environmental challenges. Among these, the proliferation of environmental endocrine disruptors (EEDs) has emerged as a critical issue, particularly in recent decades (Narváez et al., 2019; Yang et al., 2017; Wu et al., 2016). EEDs, prevalent in natural water bodies, pose serious threats to the reproductive systems and genetic integrity of both humans and wildlife (Yang et al., 2017; Wu et al., 2016; Nie et al., 2019; Wang et al., 2019). Bisphenol A (BPA), one of the most widely used industrial compounds globally, exemplifies this issue. Integral to the production of polycarbonate plastics and epoxy resins, BPA is commonly found in everyday items such as baby bottles, food packaging, and medical equipment. Approximately 27 million tons of BPA-containing plastics are produced globally each year. However, BPA’s notoriety arises from its potential to disrupt endocrine functions, posing substantial health risks, particularly to vulnerable populations like fetuses and children (Nie et al., 2019; Wang et al., 2019; Zhang et al., 2019; Zhang et al., 2018). In recognition of these dangers, the European Union has enacted a ban on BPA in baby bottles, effective 2 March 2011, underscoring the urgent need for effective remediation strategies (Qiu et al., 2015; Selvakumar et al., 2019).

In response to the growing awareness of BPA’s harmful effects, innovative degradation technologies have gained traction, with photocatalytic degradation driven by solar energy emerging as a promising approach (Yang et al., 2016; Aziz, 2019; Aziz et al., 2017; Aziz et al., 2018). Traditional metal oxide photocatalysts such as zinc oxide (ZnO) and titanium dioxide (TiO_2_) have been explored for their photocatalytic properties (Zhao et al., 2019; Zhang et al., 2014; Wu et al., 2017; Li D. et al., 2019; Zheng et al., 2020); however, their efficacy remains limited due to restricted light absorption (primarily in the UV spectrum) and rapid charge recombination (Rehman et al., 2009). This necessitates the search for alternative materials capable of efficiently harnessing visible light for BPA degradation.

Graphitic carbon nitride (g-C_3_N_4_) has attracted attention for its advantageous characteristics, including thermal stability and visible light absorbance (Wang et al., 2009a; Wang et al., 2009b; Li et al., 2011; Zhang et al., 2012; Zheng et al., 2014; Liu et al., 2015; Wang W. et al., 2024). Additionally, g-C_3_N_4_ possesses a highly negative conduction band potential (around −1.0 V vs. NHE), enhancing its ability to produce superoxide radicals for pollutant degradation (Gao et al., 2021). Nevertheless, its photocatalytic activity is often impeded by rapid charge carrier recombination and insufficient oxidizing potential (Liang et al., 2018). To address these limitations, various strategies; such as the development of heterojunctions, doping, noble metal deposition, and vacancy engineering have been employed to enhance g-C_3_N_4_ performance (Tan et al., 2022; Wang T. et al., 2024; Li et al., 2021; Cheng et al., 2023). Despite these efforts, there remains a pressing need for novel materials that exhibit high activity and stability for BPA degradation under visible light.

Recent advancements in ternary sulfides highlight the potential of materials like Zn_5_In_2_S_8_ (ZIS), which demonstrates promising photocatalytic performance (Kalomiros et al., 1987; Machuga et al., 2000; Shen et al., 2010; Wu et al., 2019; Lin et al., 2019; Liu et al., 2021; Du et al., 2023; Meng et al., 2021; Li X. et al., 2019; Zhang et al., 2024; Sharma et al., 2024; Che et al., 2024; Wan et al., 2022; Akter et al., 2024). With its lamellar structure, excellent stability, low toxicity, tunable band structure, and exceptional optoelectronic properties (Machuga et al., 2000), ZIS has shown admirable results in the fields of photocatalytic H_2_ production, H_2_O_2_ generation, and CO_2_ reduction (Shen et al., 2010; Du et al., 2023). In addition to that, ternary metal sulfide presents great potential in dealing with the most common issue of charge recombination (Li X. et al., 2019). However, its application in the photocatalytic degradation of BPA remains largely unexplored. In light of this background, this study investigates the preparation of a 2D-3D nanosheet-stacked spherical ZIS photocatalyst via a simple hydrothermal method. We compare its photoelectric properties and photocatalytic activity against g-C_3_N_4_ for effective BPA degradation. Preliminary findings indicate that ZIS is a promising candidate for tackling the persistent issue of BPA pollution through visible-light-driven photocatalysis.

## 2 Materials and methods

### 2.1 Materials

Indium chloride (InCl_3_·4H_2_O, ≥99.99%), zinc sulfate heptahydrate (ZnSO_4_·7H_2_O, ≥99.5%), urea (CO(NH_2_)_2_, ≥99.99%) thioacetamide (TAA, ≥99.0%), 5,5-dimethyl-1-pyrroline-N-oxide (DMPO, ≥98%), 2,2,6,6-Tetramethylpiperidine-1-oxyl (TEMPO, 97%), hexadecyl trimethyl ammonium bromide (CTAB, 99%), ammonium oxalate (OA, ≥99.8%), bisphenol A (BPA, ≥99%), sodium sulfate (Na_2_SO_4_, ≥99%), benzoquinone (BQ, ≥99%), potassium chloride (KCl, ≥99.5%), isopropyl alcohol (IPA, ≥99.7%), barium sulfate (BaSO_4_, ≥99.99%) and methyl viologen dichloride (MVCl_2_, ≥99%) were purchased from Sinopharm Chemical Reagent Co., Ltd. and directly used.

### 2.2 Preparation of 2D g-C_3_N_4_ and 2D-3D Zn_5_In_2_S_8_

To synthesize 2D g-C_3_N_4_ (CN), place 10 g CO(NH_2_)_2_ in the crucible, and then calcine 773.15 K for 4 h 2D-3D Zn_5_In_2_S_8_ (ZIS) was synthesized by the modification of previous studies (Shen et al., 2010; Du et al., 2023). Specifically, 1.438 g of ZnSO_4_·7H_2_O, 0.586 g of InCl_3_·4H_2_O, 0.650 g of CTAB and a double excess of TAA were dissolved in deionized water (70 mL). The solution is to be transferred into a 100 mL hydrothermal reactor, with the temperature being maintained at 433.15 K for a period of 12 h. Subsequently, the yellowish-white precipitate is to be collected and washed with deionized water and ethanol. Finally, the precipitate is to be dried in a vacuum oven at 333.15 K, resulting in the formation of the 2D-3D ZIS (Supplementary Figure S1).

### 2.3 Photocatalytic activity test

A total of 30 mg of sample powders was dispersed in 30 mL BPA solution (20 ppm BPA). This solution was stirred in darkness for 30 min to ensure equilibrium between desorption and adsorption. A 300 W Xe lamp, placed in a circular hollow chamber with water flowing through the annular casing, served as the visible light source (λ > 400 nm). At specified time intervals, 3 mL of the solution was extracted for analysis. The residual pollutants in the solution were measured using a UV-Vis spectrometer (Shimadzu UV 3600). The degradation efficiency (DE) was measured by using the formula: DE = (1 –Ct/Co) × 100%, where C_t_ and C_o_ are the BPA concentration after illumination time t and initial concentration of reactant, respectively. The rate of photocatalytic degradation (k) was assumed to follow pseudo-first-order kinetics, represented by the equation: C_t_ = C_o_e^-kt^.

### 2.4 Characterization

The morphologies were analyzed using a transmission electron microscope (TEM, FEI Tecnai G2 F20) and a field emission scanning electron microscope (FESEM, Regulus 8200). Elemental mappings were conducted with an energy-dispersive X-ray spectrometer (EDX) attached to the SEM. X-ray diffraction (XRD) patterns were obtained using a Bruker D8 X-ray powder diffractometer with Ni-filtered Cu Kα radiation. X-ray photoelectron spectroscopy (XPS) measurements were performed on a Thermo Scientific ESCA Lab250 spectrometer with an Al Kα source. UV-vis diffuse reflectance spectra (DRS) of the samples were recorded on a UV-vis spectrophotometer (Shimadzu UV-3600), with BaSO_4_ used as a reference material. Electron paramagnetic resonance (EPR) was measured using a Bruker A300. Typically, DMPO-methanol, TEMPO-water, and TEMPO-acetonitrile solution were applied to detect superoxide radicals, photoexcited electrons, and photoexcited holes, respectively. The solution containing 2 mg catalyst was loaded into a quartz tube for EPR measurements. The photoelectrochemical (PEC) properties were conducted by using an electrochemical workstation (CHI-660E) and were evaluated through photocurrent responses (i-t), open circuit potential-time (v-t) measurements, Mott-Schottky (M-S) tests, and Nyquist plots and Bode plots of electrochemical impedance spectroscopy (EIS). During PEC tests, A 300 W Xe lamp with a cutoff filter (λ > 400 nm) served as the visible light source. Ag/AgCl with 3 M KCl was used as the reference electrode. A Pt wire was applied as the counter electrode. FTO substrate deposited by the photocatalyst served as the work electrode. Na_2_SO_4_ aqueous solution (0.2 M) was used as the electrolyte for photocurrent, Mott-Schottky (M-S) plots, and photovoltage tests. KCl (0.1 M) contained K_3_ [Fe(CN)_6_]/K_4_ [Fe(CN)_6_] (0.01 M) aqueous solution was used for measuring electrochemical impedance spectroscopy (EIS) tests.

## 3 Results and discussion

### 3.1 Catalysts characterization

**FIGURE 1:**
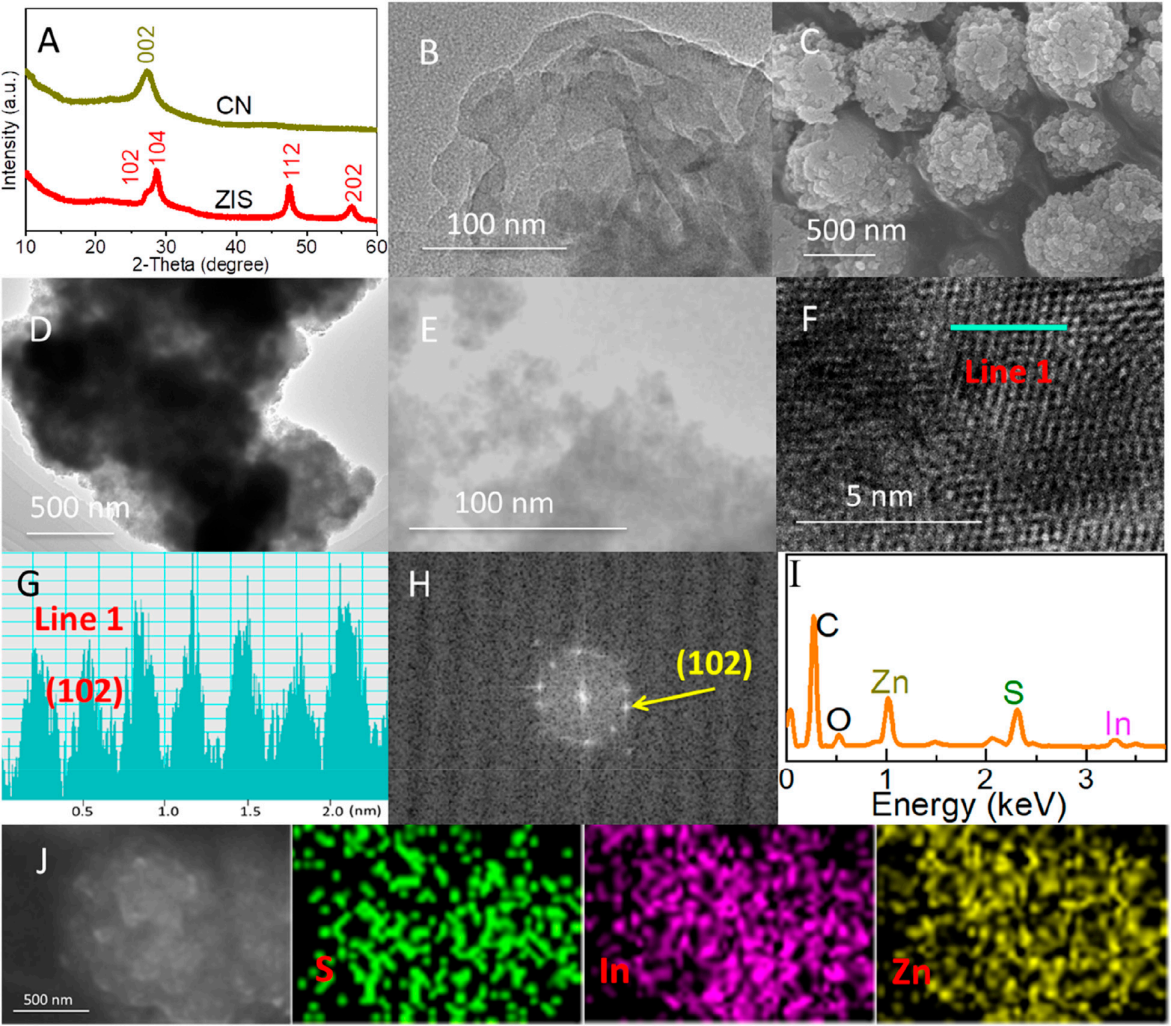
**(A)** XRD patterns of ZIS and CN. **(B)** TEM image of CN. **(C)** FESEM, **(D, E)** TEM, **(F, G)** HRTEM, **(H)** FFT, **(I, J)** EDX spectrum and element mapping images of ZIS.

The crystal phase structures of carbon nitride (CN) and zinc indium sulfide (ZIS) were analyzed by using X-ray diffraction (XRD). As presented in Figure 1A, the characteristic peak at 27.3° can be assigned to the (002) plane of graphitic carbon nitride (g-C_3_N_4_) (Wang et al., 2009b). For ZIS, the diffraction peaks at 27.2°, 28.6°, 47.6°, and 56.3° were indexed to the (102), (104), (112), and (202) lattice planes of Zn_5_In_2_S_8_, respectively (Shen et al., 2010; Du et al., 2023). The XRD spectra of both CN and ZIS are broadened, indicating their nano-structured nature. Based on the Debye-Scherrer formula (D = Kγ/Bcosθ. D, K, B, θ, and γ are the average grain size, Scherrer constant, half-height width of the diffraction peak of the measured sample, Bragg angle, and X-ray wavelength (1.54 Å), respectively.) (Akter et al., 2024), the average grain sizes of CN and ZIS were calculated to be about 10.0 nm and 12.7 nm, respectively. In addition, the dislocation density (δ) could also be obtained by the formula δ = 1/D^2^ (Akter et al., 2024). The dislocation densities of CN and ZIS were calculated to be about 1.0 × 10^−2^ and 6.2 × 10^−3^, respectively. These results indicate that CN and ZIS have fewer structural defects, and ZIS exhibits good crystal quality (the characterization results below will also prove it).

The nano-structural characteristics of CN and ZIS were visually observed by using Field Emission Scanning Electron Microscopy (FESEM) and Transmission Electron Microscopy (TEM). As presented in Figure 1B, CN displays a 2D nanosheet-like morphology, consistent with previous reports (Wang W. et al., 2024; Gao et al., 2021). Figure 1C shows the FESEM image of ZIS, exhibiting a 3D spherical structure with a rough surface. Further investigation through TEM images (Figures 1D, E) revealed that the 3D sphere of ZIS is composed of multiple thin nanosheets. The lattice fringes are clearly visible in the high-resolution TEM (HRTEM) image (Figure 1F). The lattice fringe of 0.32 nm (Figure 1G) is attributed to the (102) lattice plane of ZIS (Du et al., 2023). Additionally, the crystallographic characteristics can be verified by using the Fast Fourier Transform (FFT) pattern (Figure 1H). Energy-dispersive X-ray Spectroscopy (EDS) was carried out to investigate the constituent elements and their distribution in ZIS. As depicted in Figures 1I, J, the components of ZIS include Zn, In, and S, while C and O originate from the graphite conductive adhesive. EDS mapping confirms the uniform dispersion of indium (In), zinc (Zn), and sulfur (S) elements within the 2D-3D ZIS structure.

**FIGURE 2:**
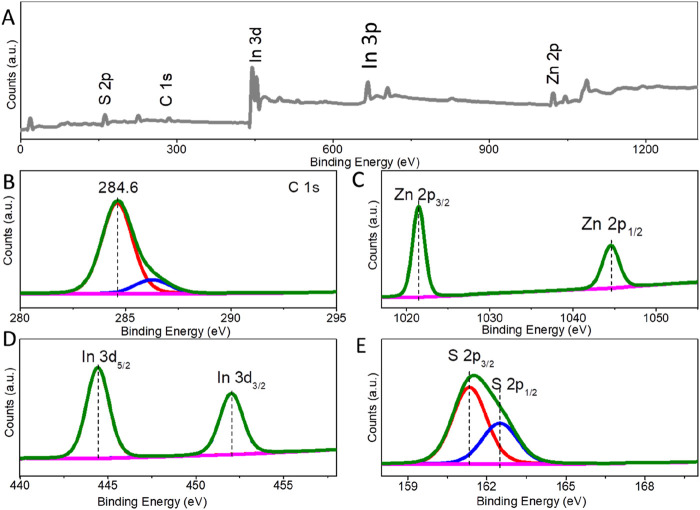
**(A)** Survey, **(B)** C 1s, **(C)** Zn 2p, **(D)** In 3d and **(E)** S 2p XPS spectra of ZIS.

To further examine the composition and elemental valence of ZIS, X-ray Photoelectron Spectroscopy (XPS) was conducted. Figure 2A is the survey spectrum of ZIS, confirming the presence of indium (In), zinc (Zn), and sulfur (S) elements. The C element and C-O species (286.2 eV) are attributed to the graphite conductive adhesive (Li X. et al., 2019). The binding energies of the constituent elements were adjusted based on the C 1s peak at 284.6 eV (Figure 2B). The fine-structured Zn 2p spectrum was deconvoluted into two peaks at approximately 1,044.6 eV and 1,021.5 eV (Figure 2C), which can be assigned to Zn 2p_1/2_ and Zn 2p_3/2_, respectively (Liu et al., 2021; Du et al., 2023). In addition, the splitting energy between 2p_1/2_ and 2p_3/2_ is 23.1 eV, indicating a Chemical valence of Zn^2+^ in the ZIS photocatalyst. The fine-structured XPS spectrum for In species shows peaks at binding energies of 452.0 eV and 444.4 eV (Figure 2D), which can be indexed to In 3d_3/2_ and In 3d_5/2_, respectively (Liu et al., 2021; Du et al., 2023). The splitting energy between 3d_5/2_ and 3d_3/2_ is 7.6 eV, which corresponds to the In^3+^ state in the ZIS. The binding energy peaks for S 2p are observed at about 161.3 eV and 162.5 eV (Figure 2E), with a splitting energy of 1.2 eV, associated with S^2-^ 2p_3/2_ and S^2-^ 2p_1/2_, respectively (Liu et al., 2021; Du et al., 2023). Thus, the above analysis indicates that a 2D-3D hierarchical structure of ZIS nanosheets and spheres has been successfully synthesized.

### 3.2 Optical properties and band energy structures

**FIGURE 3:**
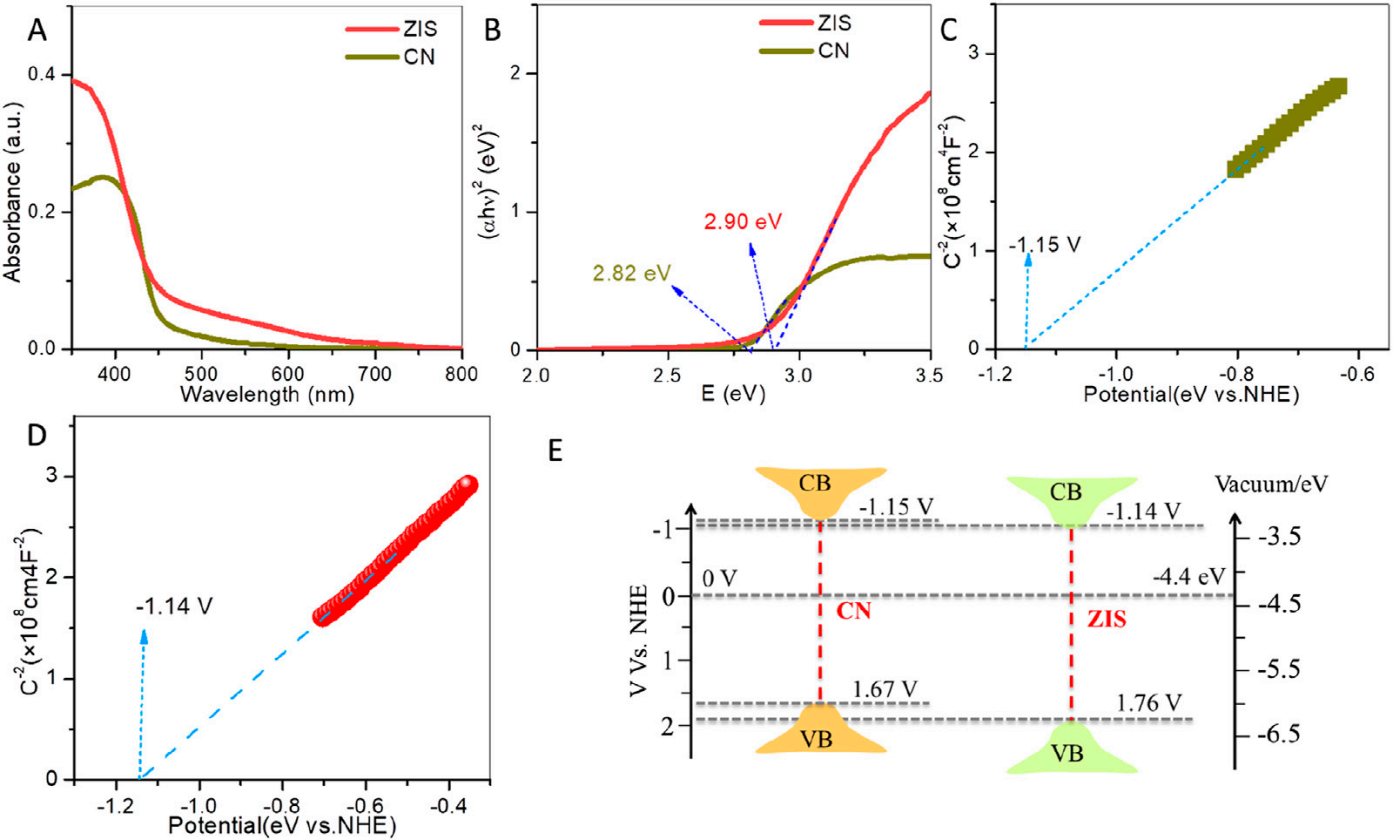
**(A)** UV-vis light absorption spectra and **(B)** Tauc plots of ZIS and CN. M-S plots (measured at 900 Hz) of **(C)** CN and **(D)** ZIS. **(E)** Band energy positions of ZIS and CN.

CN and ZIS’s optical properties and band energy structures were studied using UV-vis diffuse reflectance spectroscopy (DRS), Tauc plots, and Mott-Schottky (M-S) tests. Light absorption spectra of CN and ZIS are displayed in Figure 3A. Compared to CN, ZIS exhibits increased light absorption intensity. The enhanced light absorption in the range of less than 450 nm falls to its intrinsic absorption, while the boosted light absorption larger than 450 nm may be attributable to the rough surface of the 2D-3D ZIS nanosheet-sphere hierarchical structure. The band gap energy (E_g_) can be obtained by the Tauc equation: α*hv* = A (*E* - E_g_)^n^. The product of *h* and *v* is energy (E = *hv*). A is the proportionality constant, *h* is the Planck constant, α is the absorption coefficient, and *v* is the light frequency (Zhang et al., 2024). The values of n equal 1/2 or 2 for semiconductors with direct absorption or indirect absorption, respectively. As depicted in Figure 3B, the band gap energies of ZIS and CN are about 2.90 eV and 2.82 eV, respectively, which are close to the reported values (Liu et al., 2015; Kalomiros et al., 1987; Du et al., 2023). On the basis of the above results, it is interesting that, compared to CN, ZIS possesses strong light absorption and wide band gap energy. High light absorption and wide band gap energy would help to improve the generation and separation of photoexcited charge carriers, respectively. To demonstrate the energy band positions [valence band (VB) and conduction band (CB)] of CN and ZIS, Mott-Schottky (M-S) measurements and the formula (E_g_ = E_VB_ - E_CB_) could be used. Firstly, the flat band (FB) could be obtained by the M-S formula: 1/C^2^ = 2(V–V_FB_–kT/e)/(N_D_eεε_0_A^2^) (Sharma et al., 2024). C, V, V_FB_, T, A, k, e, N_D_, ε, and ε_0_ are the capacitance, applied potential, FB potential, absolute temperature, area, Boltzmann constant, electron charge, carrier density, dielectric constant and vacuum permittivity, respectively. For n-type semiconductors, the Fermi level (FB) is typically about 0.1 V below the conduction band (CB) (Che et al., 2024). As illustrated in Figures 3C, D, the slopes of the Mott-Schottky (M-S) plots for both CN and ZIS are positive. The results demonstrate that both ZIS and CN are n-type semiconductors. The FB values for CN and ZIS are approximately −1.05 V and −1.04 V (vs. NHE), respectively. Consequently, the CB potentials for CN and ZIS are about −1.15 V and −1.14 V, respectively, while the valence band (VB) potentials are around 1.67 V for CN and 1.76 V for ZIS (as shown in Figure 3E). The CB potential of ZIS is similar to that of CN and is more negative than the potential for producing superoxide radicals from oxygen (E (O_2_/•O_2_^−^) = −0.33 V) (Che et al., 2024). These results indicate that the reduction potential of photogenerated electrons in the CB of ZIS is comparable to that of CN, allowing for the reduction of oxygen molecules to generate superoxide radicals, which are active species involved in photocatalytic degradation. Although the oxidizing potentials of photoexcited holes in both CN and ZIS are insufficient to produce hydroxyl radicals (with an energy of E (•OH/H_2_O) = 2.40 V) (Che et al., 2024), the oxidizing potential of the photoexcited holes in the VB of ZIS is stronger than that of CN. Given these results, ZIS has stronger light absorption, larger band gap energy, and a higher VB potential compared to CN, facilitating greater photoexcited charge production, better charge separation, and enhanced degradation of BPA.

### 3.3 Optoelectronic properties

**FIGURE 4:**
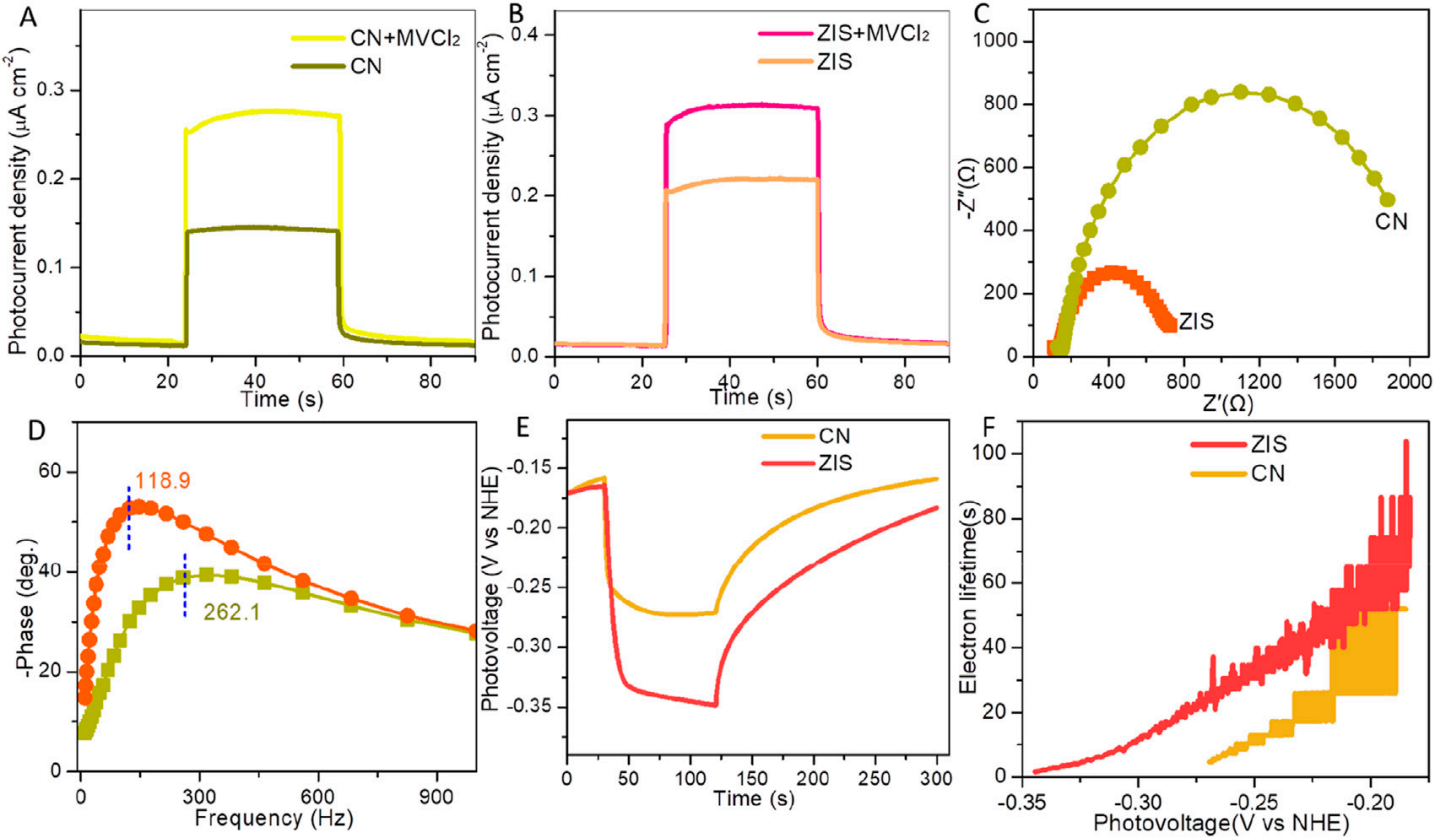
Photocurrent densities of **(A)** CN and **(B)** ZIS with and without adding MVCl_2_. EIS **(C)** Nyquist and **(D)** Bode plots of CN and ZIS. **(E)** Open circuit photovoltages and **(F)** electron lifetimes of CN and ZIS.

The photoelectrical properties of CN and ZIS were examined through a series of photoelectrochemical tests, including photocurrent measurements, electrochemical impedance spectroscopy (EIS), and photovoltage assessments. As illustrated in Figures 4A,B, the photocurrent density of ZIS is greater than that of CN under visible light irradiation. This observation indicates a higher yield and more efficient separation and transport of photoexcited electron-hole pairs. Specifically, the charge separation and transport efficiency (η) can be evaluated by using the empirical formula: η = J_H2O_/J_MVCl2_ (Wan et al., 2022). J_H2O_ and J_MVCl2_ are photocurrent densities of a photocatalyst without and with the addition of methyl viologen dichloride (MVCl_2_), respectively. As expected, ZIS (η(ZIS) = 70.8%) exhibits higher separation-transport efficiency than CN (η(CN) = 52.2%). The charge separation-transport efficiency of ZIS is about 1.356 times of CN. Figure 4C exhibits EIS Nyquist plots of ZIS and CN. The smaller arc radius means the more effective charge separation and the faster charger transportation (Meng et al., 2021). Outwardly, in contrast to CN, ZIS has a smaller arc radius, suggesting the charge separation and transfer of ZIS is superior to CN. Figure 4D is the Bode phase plots at the open-circuit voltage. According to the relationship between the lifetime of electron recombination with a time constant (t_e_) and the characteristic maximum frequency (t_e_ = 1/(2πf_max_)) (Zhou et al., 2014), the characteristic maximum frequency peak (f_max_) of ZIS (∼118.9 Hz) decrease obviously with respect to CN (∼262.1 Hz), manifesting that ZIS possesses about 2.2 times enhancement of electron lifetime than CN. Consequently, a low recombination rate of photoexcited charge carriers is highly desired to achieve in ZIS. To investigate the origin of the improved photoelectrochemical performance of ZIS, we conducted open circuit photovoltage decay (OCPV) measurements. The OCPV technique is utilized to study the lifetime of photoelectrons and the recombination rate of photoexcited charge carriers (Ning et al., 2018). As shown in Figure 4E, ZIS exhibits a higher photovoltage response compared to CN. This result aligns with the findings from photocurrent response and electrochemical impedance spectroscopy (EIS) tests, further confirming the efficient charge separation and transport in ZIS. To gain more insights, we evaluated the average photoelectron lifetime (τ_a_) using a specific equation: τ_a_ = (k_B_T/e)/(dV_OC_/dt) (Ning et al., 2018). T, k_B_, e, and t are the temperature, Boltzmann constant (1.380610 × 10^−23^ J K^−1^), electron charge (1.602 × 10^−19^ C), and time, respectively. Obviously, ZIS possesses a significantly prolonged lifetime of photoexcited electrons in comparison with CN (Figure 4F), thus contributing to the remarkably improved photoelectrochemical properties. These results imply that the photocatalytic performance of ZIS would be superior to CN.

### 3.4 Photocatalytic performance

**FIGURE 5:**
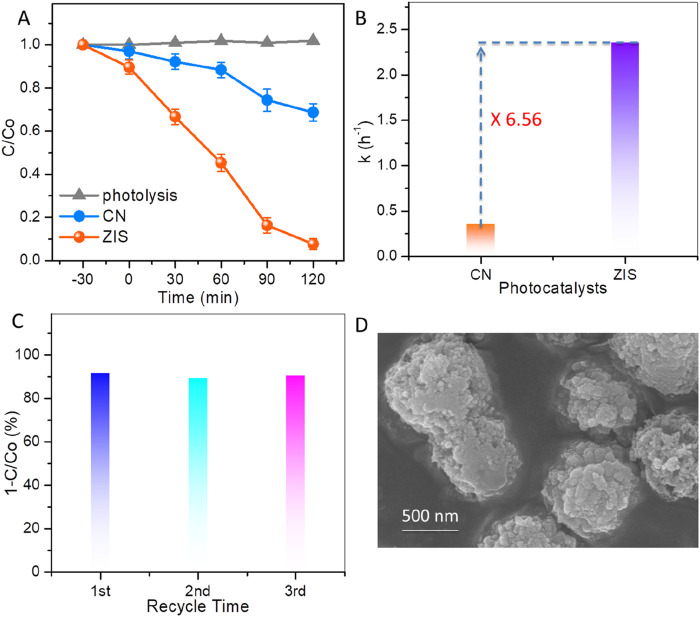
**(A)** BPA concentration changes with light irradiation time. **(B)** Kinetic reaction rate constants of BPA degradation over CN and ZIS. **(C)** Recycle experiments for photocatalytic degradation of BPA by using ZIS. **(D)** SEM image of ZIS after recycle experiment.

The performances of CN and ZIS for the photocatalytic degradation of BPA under visible light illumination are illustrated in Figure 5. To study the photocatalytic performance of the catalysts, initial tests for blank photolysis (without catalysts) and dark adsorption were conducted. As shown in Figure 5A, the results of the photolysis demonstrate that the photo-induced self-degradation of BPA is negligible, which aligns with the persistent nature of BPA. On the other hand, the dark adsorption results indicate that ZIS exhibits superior adsorption capabilities compared to CN, likely due to the 2D-3D hierarchical structure of ZIS. Under visible light irradiation, the concentration of BPA decreases rapidly, with a reduction of nearly 92.3% after 120 min of illumination. In comparison, BPA removal efficiencies of CN (Figure 5A), ZnIn_2_S_4_ (Supplementary Figure S2), and In_2_S_3_ (Supplementary Figure S2) are only 31.3%, 72.6%, and 40.4%, respectively. In addition, the BPA removal efficiency of ZIS is superior to that of the majority of the reported catalysts (Supplementary Table S1). The remarkable reactivity of ZIS is further supported by a comparison of the apparent reaction rate constants (denoted as k). The linear relationship of ln (C_0_/C) versus time (t) suggests that the degradation of BPA follows pseudo-first-order reaction kinetics (Supplementary Figure S3). The k values for CN and ZIS are approximately 0.36 h^-1^ and 2.36 h^-1^, respectively (as shown in Figure 5B). The k value for ZIS is 6.56 times higher than that for CN, highlighting ZIS’s superiority in photocatalytic degradation of BPA. Moreover, the total organic carbon (TOC) results showed that the mineralization rate of BPA over ZIS can reach about 63.5% under visible light irradiation for 120 min (Supplementary Figure S4). To confirm the stability and reusability of this photocatalyst, recycling experiments were conducted using ZIS for the photocatalytic degradation of BPA. As illustrated in Figure 5C, the photodegradation efficiency for BPA over reused ZIS remains comparable to that over fresh ZIS after three cycles. Additionally, the morphology (Figure 5D), the XRD pattern (Supplementary Figure S5), and the light absorption spectrum (Supplementary Figure S6) of the used ZIS are similar to that of the fresh ZIS, indicating that no significant changes occurred before and after the photocatalytic reaction. Thus, ZIS proves to be a stable and promising photocatalyst for the degradation of BPA.

### 3.5 Photocatalytic mechanism

**FIGURE 6:**
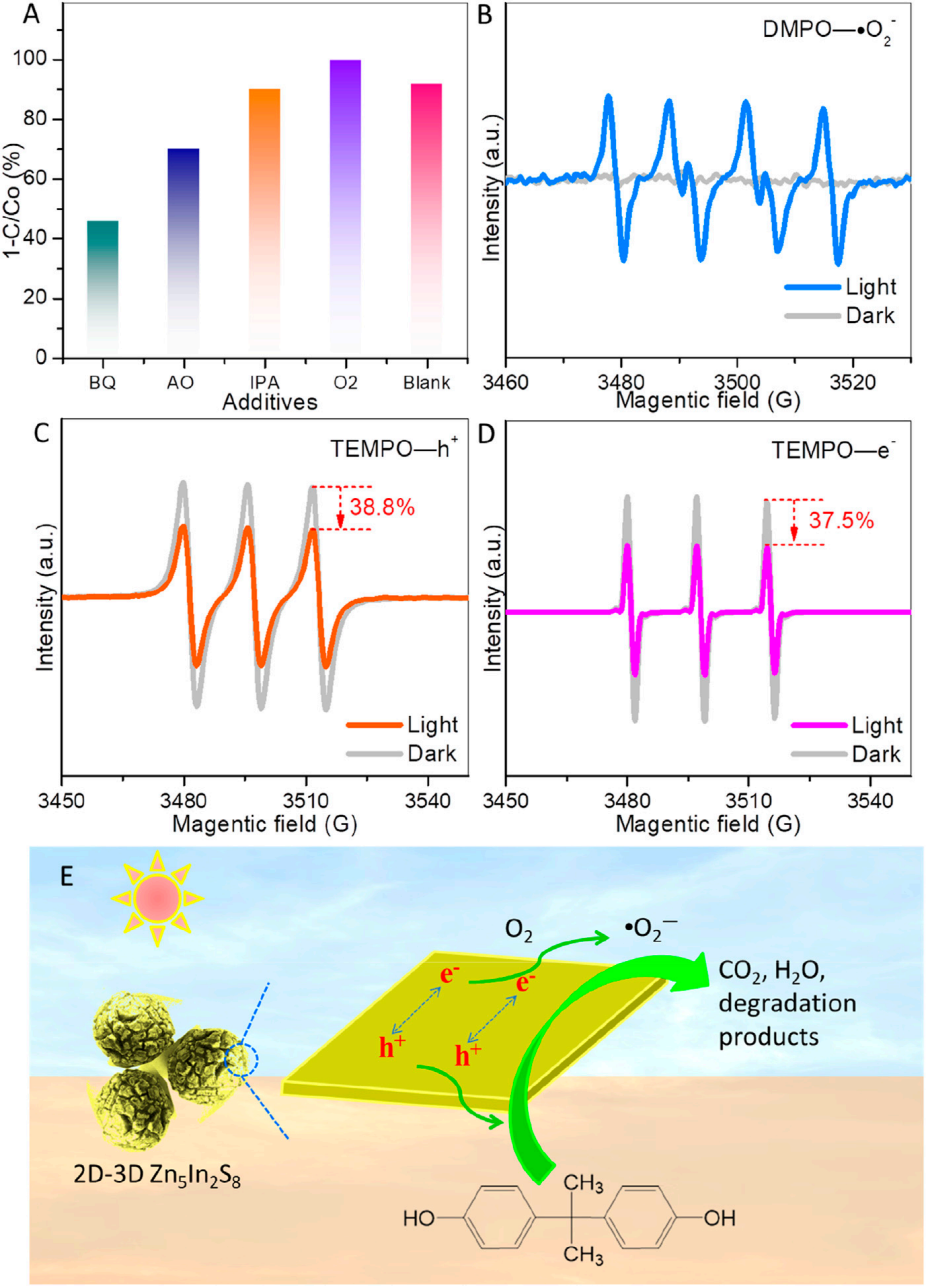
**(A)** Effects of scavengers on photocatalytic BPA degradation. **(B)** Superoxide radical (•O2–), **(C)** photoexcited hole (h+), and **(D)** photoexcited electron (e−) detection over ZIS by EPR technology (light irradiation time is 10 min). **(E)** Schematic diagram of a photocatalytic mechanism for BPA degradation on the 2D-3D spherically hierarchical structure of ZIS.

In the context of photocatalytic degradation of organic pollutants, hydroxyl radicals (•OH), photogenerated electrons (e⁻), superoxide radicals (•O_2_⁻), and photoexcited holes (h^+^) are considered active species (Song et al., 2022; Habibi Zare and Mehrabani-Zeinabad, 2023). A scavenger study was conducted to demonstrate the roles of these active species and their effect on ZIS for BPA degradation. Benzoquinone (BQ), ammonium oxalate (AO), isopropanol (IPA), and oxygen (O_2_) serve as trapping agents for •O_2_⁻, photogenerated h^+^, •OH, and photogenerated e⁻, respectively (Song et al., 2022; Habibi Zare and Mehrabani-Zeinabad, 2023). As shown in Figure 6A, the degradation efficiency of BPA is obviously inhibited by the addition of BQ (decreasing to 46.4%) and AO (decreasing to 70.3%), while IPA has minimal impact on BPA degradation. These results indicate that photogenerated holes and superoxide radicals play more critical roles than hydroxyl radicals in the degradation of BPA over ZIS. The limited effect of IPA is expected since ZIS does not generate hydroxyl radicals. Notably, when oxygen is introduced into the reaction system, almost complete degradation efficiency is achieved (99.9%). Oxygen can trap electrons, which not only creates superoxide radicals but also enhances the separation of photoexcited charges.

To verify the generation of superoxide radicals and charge separation, electron paramagnetic resonance (EPR) technology was employed (Che et al., 2024; Lei et al., 2024), as presented in Figures 6B–D. Figure 6B demonstrates that under visible light irradiation, characteristic sextet peaks of the DMPO-•O_₂⁻_ adduct can be observed, demonstrating the generation of superoxide radicals on the surface of ZIS. Additionally, TEMPO shows triplet EPR peaks. The intensity of the TEMPO EPR signal decreases when photoexcited electrons or holes are captured by TEMPO (Lei et al., 2024). Hence, TEMPO is utilized to detect photoexcited electrons and holes, and a reduction in intensity signifies photogenerated charge separation. As presented in Figures 6C, D, the TEMPO EPR intensities for holes and electrons are reduced by about 38.8% and 37.5% under light irradiation for 10 min, respectively. The TEMPO EPR peaks diminish upon detecting photoexcited holes and electrons, indicating that photoexcited charge carriers in ZIS can be effectively separated and transported to the surface for the target redox reactions.

In light of the results and analyses above, a photocatalytic mechanism for ZIS is proposed and illustrated in Figure 6E. The unique 2D-3D hierarchical structure of ZIS, with its rough surface, enhances light absorption and generates electron-hole pairs when exposed to visible light. The separated holes can directly participate in BPA degradation, while the separated electrons are captured by oxygen molecules to produce superoxide radicals, which also play a role in the degradation process of BPA.

## 4 Conclusion

In this study, we have successfully developed a 2D-3D spherically hierarchical Zn_5_In_2_S_8_ photocatalyst, marking its novel application for the photocatalytic degradation of bisphenol A (BPA). Our results indicate that Zn_5_In_2_S_8_ exhibits significantly enhanced optical and electrical properties compared to the conventional g-C_3_N_4_ photocatalyst, leading to superior performance in BPA degradation under visible light irradiation. Specifically, BPA degradation rates reached 92.3% in air and an impressive 99.9% in an oxygen-rich atmosphere after just 2 h of visible light exposure. The photodegradation of BPA follows pseudo-first-order kinetics, with Zn_5_In_2_S_8_ achieving an apparent reaction rate constant as high as 2.36 h⁻^1^, which is 6.56 times greater than that of g-C_3_N_4_. The degradation mechanism involves active species such as superoxide radicals and photogenerated holes, which play crucial roles in facilitating the breakdown of BPA. This research contributes valuable insights into the development of efficient and stable photocatalysts and underscores the potential of Zn_5_In_2_S_8_ for various photocatalytic applications, including hydrogen evolution, CO_2_ reduction, and the synthesis of hydrogen peroxide. Future investigations may focus on optimizing the performance of Zn_5_In_2_S_8_ through strategies such as vacancy engineering, heterojunction formation, and other innovative approaches. Such studies are essential for advancing the design and functionality of ternary sulfides-based photocatalysts in environmental remediation and renewable energy applications.

## Data Availability

The original contributions presented in the study are included in the article/Supplementary Material, further inquiries can be directed to the corresponding authors.

## References

[B1] Akter S. Sikdar T. T. Sultana M. Ahmed S. Bashar M. S. Rahman M. K. (2024). Enhancing the performance of CuO thin film in solar cell by introducing optimum amount of Ni doping. J. Mater. Sci. Mater. Electron. 35 (19), 1299–1314. 10.1007/s10854-024-13053-x

[B2] Aziz K. H. H. (2019). Application of different advanced oxidation processes for the removal of chloroacetic acids using a planar falling film reactor. Chemosphere 228, 377–383. 10.1016/j.chemosphere.2019.04.160 31042611

[B3] Aziz K. H. H. Miessner H. Mueller S. Kalass D. Moeller D. Khorshid I. (2017). Degradation of pharmaceutical diclofenac and ibuprofen in aqueous solution, a direct comparison of ozonation, photocatalysis, and non-thermal plasma. Chem. Eng. J. 313, 1033–1041. 10.1016/j.cej.2016.10.137

[B4] Aziz K. H. H. Miessner H. Mueller S. Mahyar A. Kalass D. Moeller D. (2018). Comparative study on 2,4-dichlorophenoxyacetic acid and 2,4-dichlorophenol removal from aqueous solutions via ozonation, photocatalysis and non-thermal plasma using a planar falling film reactor. J. Hazard. Mater. 343, 107–115. 10.1016/j.jhazmat.2017.09.025 28942183

[B5] Che Y. Weng B. Li K. He Z. Chen S. Meng S. (2024). Chemically bonded nonmetallic LSPR S-scheme hollow heterostructure for boosting photocatalytic performance. Appl. Catal. B Environ. Energy 361, 124656. 10.1016/j.apcatb.2024.124656

[B6] Cheng S. Sun Z. Lim K. H. Wibowo A. A. Zhang T. Du T. (2023). Dual-defective two-dimensional/two-dimensional Z-scheme heterojunctions for CO_2_ reduction. ACS Catal. 13 (11), 7221–7229. 10.1021/acscatal.3c00219

[B7] Du Z. Gong K. Yu Z. Yang Y. Wang P. Zheng X. (2023). Photoredox coupling of CO_2_ reduction with benzyl alcohol oxidation over ternary metal chalcogenides (Zn_m_In_2_S_3+m_, m = 1–5) with regulable products selectivity. Molecules 28 (18), 6553. 10.3390/molecules28186553 37764329 PMC10537807

[B8] Gao S. Wang X. Song C. Zhou S. Yang F. Kong Y. (2021). Engineering carbon-defects on ultrathin g-C_3_N_4_ allows one-pot output and dramatically boosts photoredox catalytic activity. Appl. Catal. B Environ. 295, 120272. 10.1016/j.apcatb.2021.120272

[B9] Habibi Zare M. Mehrabani-Zeinabad A. (2023). Yolk@Wrinkled-double shell smart nanoreactors: new platforms for mineralization of pharmaceutical wastewater. Front. Chem. 11, 1211503. 10.3389/fchem.2023.1211503 37347043 PMC10281210

[B10] Kalomiros J. A. Anagnostopoulos A. N. Spyridelis J. (1987). Growth and some properties of Zn_5_In_2_S_8_ single crystal. Mat. Res. Bull. 22 (10), 1307–1314. 10.1016/0025-5408(87)90293-5

[B11] Lei J. Zhou N. Sang S. Meng S. Low J. Li Y. (2024). Unraveling the roles of atomically-dispersed Au in boosting photocatalytic CO_2_ reduction and aryl alcohol oxidation. Chin. J. Catal. 65, 163–173. 10.1016/S1872-2067(24)60109-9

[B12] Li D. Yan X. Yang M. Luo C. Li P. Hu J. (2019a). 4-Mercaptobenzoic acid assisted synthesis of Au-decorated alpha-Fe_2_O_3_ nanopa ticles with highly enhanced photocatalytic performance. J. Alloy. Compd. 775, 150–157. 10.1016/j.jallcom.2018.10.073

[B13] Li W. Chu X. S. Wang F. Dang Y. Y. Liu X. Y. Wang X. C. (2021). Enhanced cocatalyst-support interaction and promoted electron transfer of 3D porous g-C_3_N_4_/GO-M (Au, Pd, Pt) composite catalysts for hydrogen evolution. Appl. Catal. B Environ. 288, 120034. 10.1016/j.apcatb.2021.120034

[B14] Li X. Sun Y. Xu J. Shao Y. Wu J. Xu X. (2019b). Selective visible-light-driven photocatalytic CO_2_ reduction to CH_4_ mediated by atomically thin CuIn_5_S_8_ layers. Nat. Energy 4, 690–699. 10.1038/s41560-019-0431-1

[B15] Li X. H. Chen J. S. Wang X. Sun J. Antonietti M. (2011). Metal-free activation of dioxygen by graphene/g-C_3_N_4_ nanocomposites: functional dyads for selective oxidation of saturated hydrocarbons. J. Am. Chem. Soc. 133 (21), 8074–8077. 10.1021/ja200997a 21561075

[B16] Liang X. Wang G. Dong X. Wang G. Ma H. Zhang X. (2018). Graphitic carbon nitride with carbon vacancies for photocatalytic degradation of bisphenol A. ACS Appl. Nano Mater. 2 (1), 517–524. 10.1021/acsanm.8b02089

[B17] Lin J. Wu X. Xie S. Chen L. Zhang Q. Deng W. (2019). Visible‐light‐driven cleavage of C−O linkage for lignin valorization to functionalized aromatics. ChemSusChem 12 (22), 5023–5031. 10.1002/cssc.201902355 31583821

[B18] Liu J. Liu Y. Liu N. Han Y. Zhang X. Huang H. (2015). Metal-free efficient photocatalyst for stable visible water splitting via a two-electron pathway. Science 347 (6225), 970–974. 10.1126/science.aaa3145 25722405

[B19] Liu J. Xue X. Zhou X. Chen G. Liu W. (2021). Effect of anisotropic conductivity of Ag_2_ S-modified Zn_m_In_2_S_3+ m_ (m = 1, 5) on the photocatalytic properties in solar hydrogen evolution. RSC Adv. 11 (43), 26908–26914. 10.1039/d1ra05413a 35479976 PMC9037693

[B20] Machuga A. I. Zhitar' V. F. Muntyan S. P. Arama E. D. (2000). Growth and cathodoluminescence properties of Zn_5_In_2_S_8_ single crystals. Inorg. Mater. 36, 1192–1193. 10.1023/A:1026661010429

[B21] Meng S. Chen C. Gu X. Wu H. Meng Q. Zhang J. (2021). Efficient photocatalytic H_2_ evolution, CO_2_ reduction and N_2_ fixation coupled with organic synthesis by cocatalyst and vacancies engineering. Appl. Catal. B Environ. 285, 119789. 10.1016/j.apcatb.2020.119789

[B22] Narváez J. F. Grant H. Gil V. C. Porras J. Sanchez J. C. B. Duque L. F. O. (2019). Assessment of endocrine disruptor effects of levonorgestrel and its photoproducts: environmental implications of released fractions after their photocatalytic removal. J. Hazard. Mater. 371, 273–279. 10.1016/j.jhazmat.2019.02.095 30856437

[B23] Nie M. Deng Y. Nie S. Yan C. Ding M. Dong W. (2019). Simultaneous removal of bisphenol A and phosphate from water by peroxymonosulfate combined with calcium hydroxide. Chem. Eng. J. 369, 35–45. 10.1016/j.cej.2019.03.046

[B24] Ning X. Zhen W. Wu Y. Lu G. (2018). Inhibition of CdS photocorrosion by Al_2_O_3_ shell for highly stable photocatalytic overall water splitting under visible light irradiation. Appl. Catal. B Environ. 226, 373–383. 10.1016/j.apcatb.2017.12.067

[B25] Qiu P. Li W. Thokchom B. Park B. Cui M. Zhao D. (2015). Uniform core-shell structured magnetic mesoporous TiO_2_ nanospheres as a highly efficient and stable sonocatalyst for the degradation of bisphenol-A. J. Mater. Chem. A 3 (12), 6492–6500. 10.1039/C4TA06891B

[B26] Rehman S. Ullah R. Butt A. M. Gohar N. D. (2009). Strategies of making TiO_2_ and ZnO visible light active. J. Hazard. Mater. 170 (2-3), 560–569. 10.1016/j.jhazmat.2009.05.064 19540666

[B27] Selvakumar K. Raja A. Arunpandian M. Stalindurai K. Rajasekaran K. Sami P. (2019). Efficient photocatalytic degradation of ciprofloxacin and bisphenol A under visible light using Gd_2_WO_6_ loaded ZnO/bentonite nanocomposite. Appl. Surf. Sci. 481, 1109–1119. 10.1016/j.apsusc.2019.03.178

[B28] Sharma P. Kumar A. Dhiman P. Sharma G. Sillanpää M. Wang T. (2024). Flower shaped Zn_2_In_2_S_5_/FeIn_2_S_4_ as a promising S-Scheme heterojunction photocatalyst for superior ciprofloxacin removal. Mater. Today Commun. 39, 109051. 10.1016/j.mtcomm.2024.109051

[B29] Shen S. Zhao L. Guo L. (2010). Zn_m_In_2_S_3+m_ (m = 1–5, integer): a new series of visible-light-driven photocatalysts for splitting water to hydrogen. Int. J. Hydrogen Energy 35 (19), 10148–10154. 10.1016/j.ijhydene.2010.07.171

[B30] Song J. Zhao K. Yin X. Liu Y. Khan I. Liu S. Y. (2022). Photocatalytic degradation of tetracycline hydrochloride with g-C_3_N_4_/Ag/AgBr composites. Front. Chem. 10, 1069816. 10.3389/fchem.2022.1069816 36451930 PMC9702527

[B31] Tan M. Ma Y. Yu C. Luan Q. Li J. Liu C. (2022). Boosting photocatalytic hydrogen production via interfacial engineering on 2D ultrathin Z‐scheme ZnIn_2_S_4_/g‐C_3_N_4_ heterojunction. Adv. Funct. Mater. 32 (14), 2111740. 10.1002/adfm.202111740

[B32] Wan J. Liu L. Wu Y. Song J. Liu J. Song R. (2022). Exploring the polarization photocatalysis of ZnIn_2_S_4_ material toward hydrogen evolution by integrating cascade electric fields with hole transfer vehicle. Adv. Funct. Mater. 32 (35), 2203252. 10.1002/adfm.202203252

[B33] Wang L. Zhang Y. Liu Y. Gong X. Zhang T. Sun H. (2019). Widespread occurrence of bisphenol A in daily clothes and its high exposure risk in humans. Environ. Sci. Technol. 53 (12), 7095–7102. 10.1021/acs.est.9b02090 31124657

[B34] Wang T. Pan X. He M. Kang L. Ma W. (2024b). *In situ* construction of hollow coral‐like porous S‐doped g‐C_3_N_4_/ZnIn_2_S_4_ S‐scheme heterojunction for efficient photocatalytic hydrogen evolution. Adv. Sci. 11 (33), 2403771. 10.1002/advs.202403771 PMC11434114 38961647

[B35] Wang W. Liu R. Zhang J. Kong T. Wang L. Yu X. (2024a). Building asymmetric Zn–N3 bridge between 2D photocatalyst and Co‐catalyst for directed charge transfer toward efficient H_2_O_2_ synthesis. Angew. Chem. Int. Ed., e202415800. 10.1002/anie.202415800 39377644

[B36] Wang X. Chen X. Thomas A. Fu X. Antonietti M. (2009b). Metal‐containing carbon nitride compounds: a new functional organic–metal hybrid material. Adv. Mater. 21 (16), 1609–1612. 10.1002/adma.200802627

[B37] Wang X. Maeda K. Thomas A. Takanabe K. Xin G. Carlsson J. M. (2009a). A metal-free polymeric photocatalyst for hydrogen production from water under visible light. Nat. Mater. 8 (1), 76–80. 10.1038/nmat2317 18997776

[B38] Wu D. Tao X. Chen Z. P. Han J. T. Jia W. J. Zhu N. (2016). The environmental endocrine disruptor p-nitrophenol interacts with FKBP51, a positive regulator of androgen receptor, and inhibits androgen receptor signaling in human cells. J. Hazard. Mater. 307, 193–201. 10.1016/j.jhazmat.2015.12.045 26780698

[B39] Wu S. C. Tan C. S. Huang M. H. (2017). Strong facet effects on interfacial charge transfer revealed through the examination of photocatalytic activities of various Cu_2_O-ZnO heterostructures. Adv. Funct. Mater. 27 (9), 1604635. 10.1002/adfm.201604635

[B40] Wu Y. Wang H. Tu W. Wu S. Chew J. W. (2019). Effects of composition faults in ternary metal chalcogenides (Zn_x_In_2_S_3+x_, x = 1–5) layered crystals for visible-light-driven catalytic hydrogen generation and carbon dioxide reduction. Appl. Catal. B Environ. 256, 117810. 10.1016/j.apcatb.2019.117810

[B41] Yang H. Q. Huang Y. Liu J. Y. Tang P. X. Sun Q. M. Xiong X. N. (2017). Binding modes of environmental endocrine disruptors to human serum albumin: insights from STD-NMR, ITC, spectroscopic and molecular docking studies. Sci. Rep. 7 (1), 11126. 10.1038/s41598-017-11604-3 28894220 PMC5593971

[B42] Yang Y. Que W. Zhang X. Xing Y. Yin X. Du Y. (2016). Facile synthesis of ZnO/CuInS_2_ nanorod arrays for photocatalytic pollutants degradation. J. Hazard. Mater. 317, 430–439. 10.1016/j.jhazmat.2016.05.080 27322900

[B43] Zhang H. Gao Y. Meng S. Wang Z. Wang P. Wang Z. (2024). Metal sulfide S‐scheme homojunction for photocatalytic selective phenylcarbinol oxidation. Adv. Sci. 11 (17), 2400099. 10.1002/advs.202400099 PMC11077664 38417112

[B44] Zhang J. Zhang M. Sun R. Q. Wang X. (2012). A facile band alignment of polymeric carbon nitride semiconductors to construct isotype heterojunctions. Angew. Chem. Int. Ed. 40 (51), 10145–10149. 10.1002/anie.201205333 22961676

[B45] Zhang S. Bao J. Gong X. Shi W. Zhong X. (2019). Hazards of bisphenol A——blocks RNA splicing leading to abnormal testicular development in offspring male mice. Chemosphere 230, 432–439. 10.1016/j.chemosphere.2019.05.044 31121507

[B46] Zhang X. Liu Y. Kang Z. (2014). 3D branched ZnO nanowire arrays decorated with plasmonic au nanoparticles for high-performance photoelectrochemical water splitting. ACS Appl. Mater. Inter. 6 (6), 4480–4489. 10.1021/am500234v 24598779

[B47] Zhang Y. Cui W. An W. Liu L. Liang Y. Zhu Y. (2018). Combination of photoelectrocatalysis and adsorption for removal of bisphenol A over TiO_2_-graphene hydrogel with 3D network structure. Appl. Catal. B Environ. 221, 36–46. 10.1016/j.apcatb.2017.08.076

[B48] Zhao G. Sun Y. Zhao Y. Wen T. Wang X. Chen Z. (2019). Enhanced photocatalytic simultaneous removals of Cr (VI) and bisphenol A Over Co (II)-modified TiO_2_ . Langmuir 35 (1), 276–283. 10.1021/acs.langmuir.8b03214 30550286

[B49] Zheng X. Zhang Z. Meng S. Wang Y. Li D. (2020). Regulating charge transfer over 3D Au/ZnO hybrid inverse opal toward efficiently photocatalytic degradation of bisphenol A and photoelectrochemical water splitting. Chem. Eng. J. 393, 124676. 10.1016/j.cej.2020.124676

[B50] Zheng Y. Lin L. Ye X. Guo F. Wang X. (2014). Helical graphitic carbon nitrides with photocatalytic and optical activities. Angew. Chem. Int. Ed. 126 (44), 11926–11930. 10.1002/anie.201407319 25220601

[B51] Zhou M. Bao J. Xu Y. Zhang J. Xie J. Guan M. (2014). Photoelectrodes based upon Mo: BiVO_4_ inverse opals for photoelectrochemical water splitting. Acs Nano 8 (7), 7088–7098. 10.1021/nn501996a 24911285

