# Supplementary material for: Efficient photocatalytic degradation of bisphenol A on 2D-3D spherically hierarchical structure Zn5In2S8
Source: Front Chem. 2025 Jan 16;12:1519370. doi: 10.3389/fchem.2024.1519370 (PMC11780547; doi:10.3389/fchem.2024.1519370)
Supplement: Supplementary file 1 [file DataSheet1.pdf]

Supporting Information

Efficient photocatalytic degradation of bisphenol A on 2D-3D spherically hierarchical structure $Zn_5In_2S_8$

Zongwen Zhang [1], Yi Zhang [2,*], Huili Han [3], Riyadh Ramadhan Ikreedeegh [5], Syed Shoaib Ahmad Shah [6], Muhammad Tayyab [4,*]

[1] Analysis & Testing Center, Xinyang Normal University, Xinyang, Henan 464000, China.

[2] Department of Chemistry, Key Laboratory of Green and Precise Synthetic Chemistry, Ministry of Education, Huaibei Normal University, Huaibei, Anhui 235000, China.

[3] Collaborative Innovation Center of Henan Province for Energy-Saving Building Materials, Xinyang Normal University, Xinyang, Henan 464000, China.

[4] Institute of Materials Research, Tsinghua Shenzhen International Graduate School, Tsinghua University, Shenzhen, Guangdong 518055, China.

[5] Department of Analysis and Quality Control, Sarir Oil Refinery, Arabian Gulf Oil Company, El Kish, P.O. Box 263, Benghazi, Libya.

[6] Department of Chemistry, School of Natural Sciences, National University of Sciences and Technology, Islamabad, 44000, Pakistan.

[*] Corresponding authors, E-mail: zhangyi@chnu.edu.cn (Y. Zhang); m.tayyab72@sz.tsinghua.edu.cn (M. Tayyab).

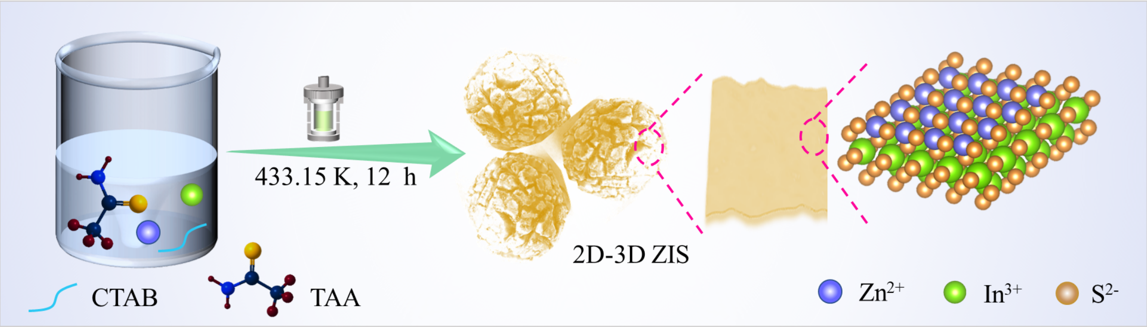

FIGURE S1

Schematic of 2D-3D ZIS nanosheet-sphere hierarchical structure and preparation process.

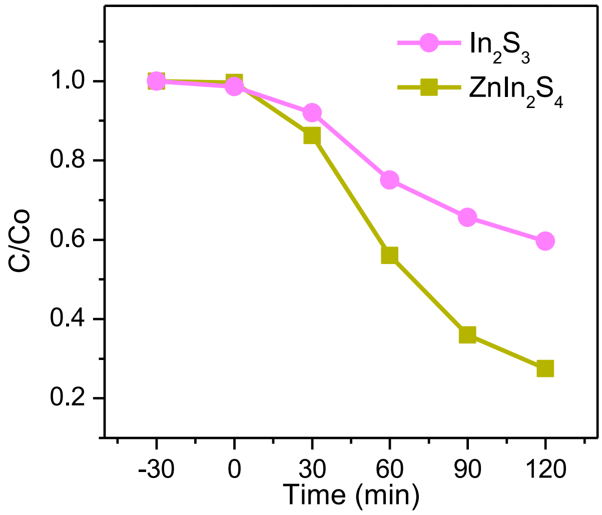

FIGURE S2

The photocatalytic activities of In_2S_3 and $ZnIn_2S_4$ for photocatalytic degradation of BPA.

In order to compare the photocatalytic activity of ZIS with that of other sulfides, we compared the activity of ZIS with that of $ZnIn_2S_4$ and In_2S_3. $ZnIn_2S_4$ and In_2S_3 were prepared by Applied Surface Science 384 (2016) 161–174 and RSC Adv., 2017, 7, 6457–6466, respectively. As shown in Figure S1, ZIS is more active than $ZnIn_2S_4$, but significantly better than In_2S_3. At the same experimental condition, BPA degradation efficiencies of ZIS, $ZnIn_2S_4$ and In_2S_3 are 92.3%, 72.6% and 40.4%, respectively.

Table S1 Comparison of ZIS with other photocatalysts for photocatalytic degradation of BPA.

Catalysts	Mass of Catalyst	BPA concentration	Reaction time	Degradation efficiency	Reference
In_2O_3	100 mg	20 ppm	180 min	~25%	Appl. Surf. Sci., 2023, 631, 157531.
CN	100 mg	20 ppm	180 min	~35%	Appl. Surf. Sci., 2023, 631, 157531.
In_2O_3/Cu-CN	100 mg	20 ppm	180 min	91%	Appl. Surf. Sci., 2023, 631, 157531.
$W_{18}O_{49}$	25 mg	10 ppm	150 min	< 55%	Colloid Surface A, 2024, 703, 135050.
PDI	25 mg	10 ppm	150 min	~55%	Colloid Surface A, 2024, 703, 135050.
Co-$W_{18}O_{49}$/PDI	25 mg	10 ppm	150 min	91.2%	Colloid Surface A, 2024, 703, 135050.
N-NiO@Zeolite	10 mg	4 ppm	120 min	94%	J. Photoch. Photobio. A, 2024, 1,115790.
2-BFO/NBWO	200 mg	20 ppm	120 min	95.5%	Colloid Surface A, 2024, 692, 133905.
0.05 OA-UN-CN	70 mg	10 ppm	180 min	100%	Green Energy Environ., 2024, 7, 1159-1170.
α-MnO_2	50 mg	20 ppm	120 min	40%	J. Environ. Chem. Eng., 2024, 12, 112879.
OCN-1.5	50 mg	30 ppm	300 min	82.55%	ACS Appl. Nano Mater., 2023, 6, 16567-16579.
c-SB/ZnO	20 mg	20 ppm	150 min	94.8%	Sci. Total. Environ., 2024, 907, 167896.
SCN/CD3	50 mg	50 ppm	120 min	90%	Mater. Adv., 2024, 5, 5514-5526.
$CeVO_4$/CN /$LaNiO_3$	20 mg	10 ppm	180 min	68%	Mater. Today Commun., 2024, 39, 108721.
BOC/BOI	30 mg	10 ppm	120 min	70%	Front. Chem., 2023, 10, 1102528.
**ZIS**	**30 mg**	**20 ppm**	**120 min**	**92.3%**	**This work**

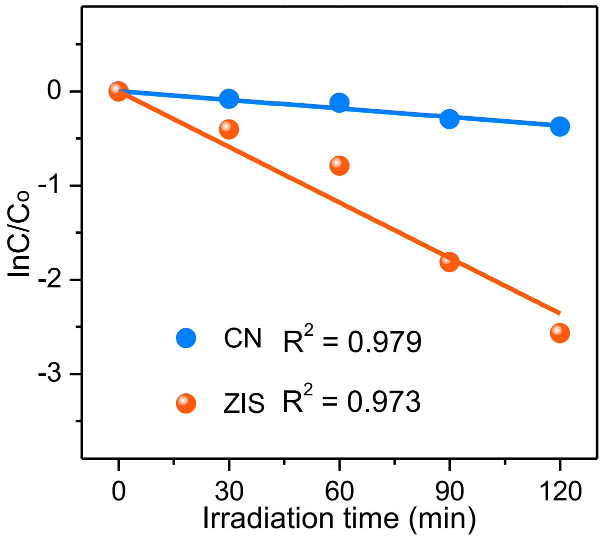

FIGURE S3

The kinetic curves (lnC/C_0 - t) of BPA degradation over CN and ZIS.

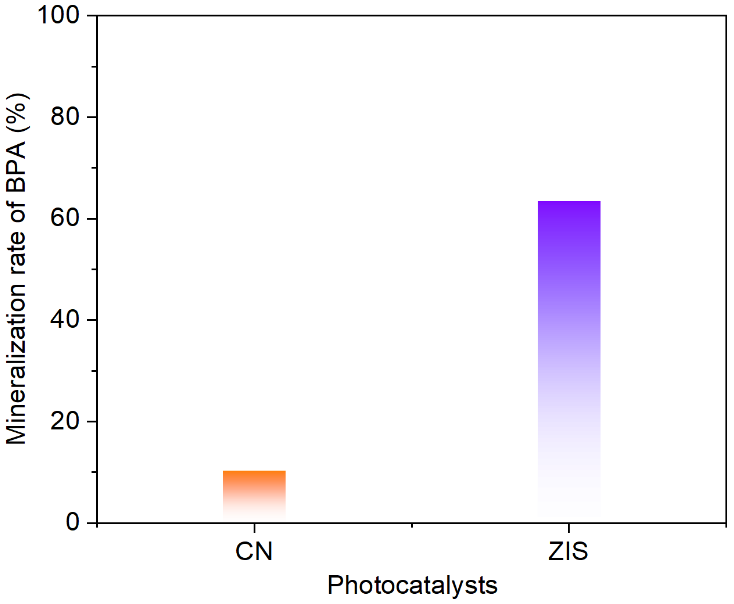

FIGURE S4

The mineralization rates of BPA over CN and ZIS under visible light irradiation for 120 min.

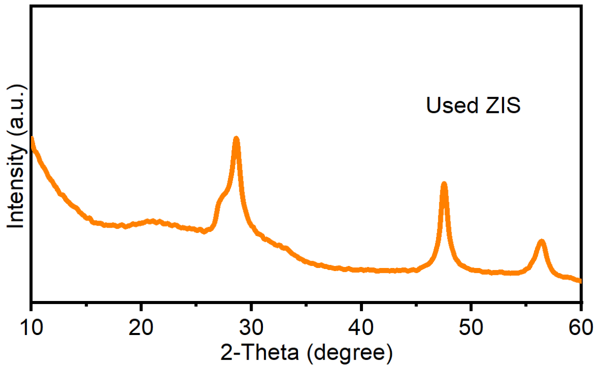

FIGURE S5

XRD pattern of the used ZIS for photocatalytic degradation of BPA.

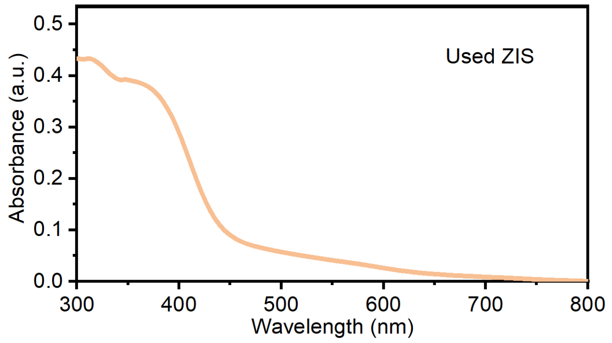

FIGURE S6

UV-vis light absorption spectrum of the used ZIS for photocatalytic degradation of BPA.
